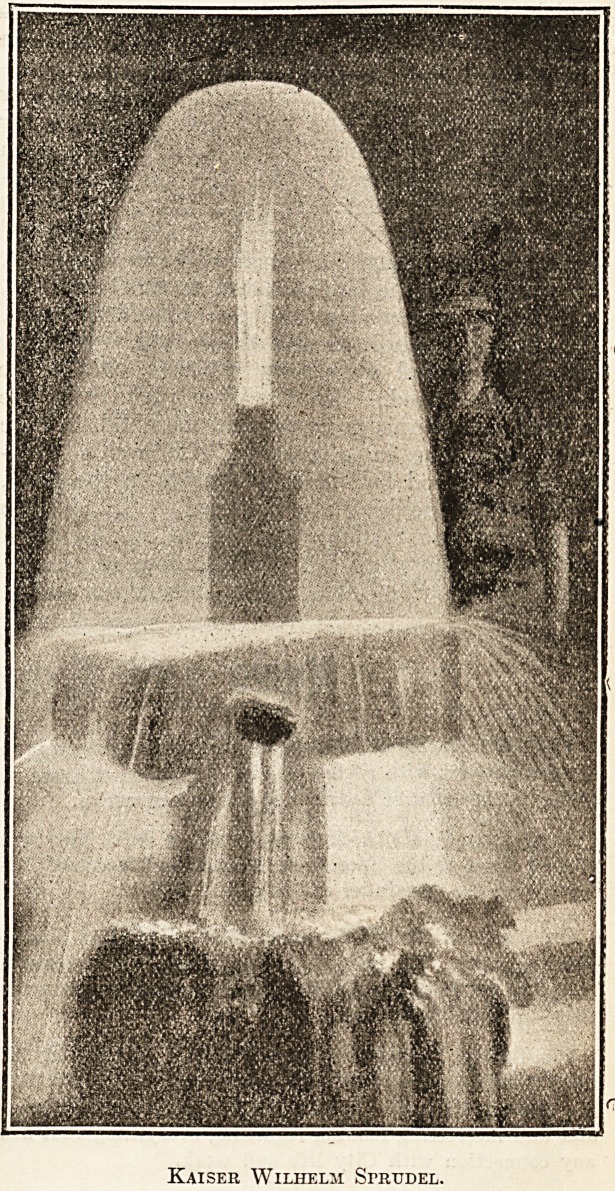# Home and Continental Spas
*Previous articles in this series appeared in The Hospital of January 28, February 25, and March 25


**Published:** 1911-04-22

**Authors:** 


### HOME AND CONTINENTAL SPAS.*

### IV.—OEYNHAUSEN.

Of the less well-known Continental spas, there are few that for attractiveness, comfort, and general all-round excellence can compare with the little Westphalian town of Oeynhausen. There is none that can surpass it for just those points that ought to make it popular with the English patient of moderate means who desires a thoroughly modern bathing-place without the annoyances which inevitably cluster round an expensive and fashionable health-resort. Oeynhausen is homely, comfortable, cheap, attractive, and enchanting. Those who have patronised it once will come back to it, for there is something in its quiet simplicity, in its unostentatious excellence, that appeals strongly to the imagination, just as its beautiful environment of hill and stream appeals to the artistic taste.

### How to Get There.

It is easily, very pleasantly, reached from London by various routes. The Bade Verwaltung, that has taken care to investigate the excellences of every route, recommends the Queenborough-Flushing express, the fares for which are, first-class return (available sixty days) £4 18s. 6d.; second-class return £3 8s. The Dover-Calais and Dover-Ostend routes, both with shorter sea-crossings, will attract those patients who are not fond of risking the discomforts of a Channel passage. We ourselves prefer, of all methods of reaching the Continent, the excellent Harwich-Hook of Holland service of the Great Eastern line, which in our opinion will be found much the most comfortable for invalids. This route is recommended also on account of its cheapness. Those who have no fear of the sea, and who have plenty of time available, may find another and comparatively little used route viâ Hamburg, by the steamers of the General Steam Navigation Company. For ordinary purposes, however, the choice will lie between the Flushing and Harwich routes.

### The Story of the Spa.

Oeynhausen as a spa dates from the early days of the last century, when an attempt to bore for mineral salt revealed the existence of a spring which possesses a temperature of 32¼ degrees Centigrade. It was well known that the water of this spring was rich in chlorides; indeed the original boring had been for a stratum of rock salt which it was hoped would rival the Neusaltzwerk Mine. The Government quickly realised the valuable asset which the possession of this spring constituted, and took means to secure it from private exploitation. The Kurhaus was placed under direct State control, the new spa was christened after its discoverer, Herr Oeynhausen, who had worked indefatigably to interest the Government in his discovery, a branch railway-line was constructed to give greater facilities to visitors, and building operations were started to bring the town in line with its rivals. In 1857 a fine thermal-bath house was opened at a cost of £15,000, and a few years later a vapour-bath house and a mineral-bath house were erected. In the early eighties additional bath-houses were provided, and in 1898 the finest spring, the magnificent Kaiser Wilhelm Sprudel, was opened up, and a bath-house worthy of the new spring was erected at a cost of £20.000 and opened two years later. Since then Oeynhausen has rapidly won fame and popularity, and it needs only an introduction to English patients to become one of the most popular of Continental spas with the British public.

### The Town and its Environment.

The town lies at an altitude of 220 feet above the sea level, and is situate in the district of Minden,

Plan of the New Kurhaus and Gardens.

on the right bank of the river Werre, a tributary of the Weser, and about six miles from the impressive "Westphalian Gate," where the latter river emerges from the mountains. It is surrounded by beautiful woods, outliers of the Teutoburg Forest, with picturesque, red-roofed farms and villages clustering around. The high Weser mountains adequately protect it from the cold winds that sweep over the Westphalian uplands,

* Previous articles in this series appeared in The Hospital of January 28, February 25, and March 25.

and it enjoys an equable climate. Itself a town of considerable beauty, with broad, clean streets which are really boulevards bordered with tall lime trees, Oeynhausen impresses the visitor at first sight by its aspect of homeliness and quiet dignity, which is in sharp contrast to the ultra-modernity and flashy attractiveness of the more fashionable German spas. The municipality has taken a paternal interest in the town, and the result is that Oeynhausen possesses a most excellent system of

The Royal Kurhaus (General View).

drainage, a perfect water-supply, and combines the artistic orderliness of a garden city with the scientific advantages of a well-arranged modern bathing establishment.

### The Springs.

There are five thermal muriated springs, varying in temperature from 55° Fahrenheit to over 92° Fahrenheit. The percentage of solids in the water averages 4.2, and the waters resemble most closely those of Bad Nauheim, surpassing these, however, in the amount of solid constituents—principally chlorides of sodium, lithium, potassium, with sulphates of these metals, and traces of arseniates and iodides. They also contain a very large amount of $CO_2$ gas, averaging 900 cc. to the litre, so that it is possible to prepare strong hot baths rich in carbonic acid simply by mixing the waters. In 1906 the fifth spring was opened up, but the finest of the five still remains, the Kaiser Wilhelm Sprudel, the boring of which goes down to a depth of nearly 2,200 feet and which cost over £13,000. The advantage which Oeynhausen possesses over its rivals, Nauheim and Kissingen, is the facility with which, by simple mixture of the waters from the various springs, strong thermal baths can be prepared without resorting to the aid of steam for heating purposes. In addition to these muriated thermal waters the spa possesses two cold-brine springs which average 10 per cent. of solid constituents, and are peculiarly rich in iodide and bromide combinations, which render them very suitable for the preparation of strong brine-baths.

### Oeynhausen Specialties.

Special arrangements exist for dealing with diseases of the nervous system, joints and bones, blood and the circulatory system, and of the mucous membranes of the respiratory and digestive tracts. To these must be added diseases of women. There is a presupposition among English medical men that the only Continental spa to which patients suffering from heart disease can be recommended is Bad Nauheim: this, in view of the excellence of the treatment of such conditions at Oeynhausen, is a mistake. The Oeynhausen waters are particularly adapted for the Nauheim treatment, and their relatively high percentage of carbonic acid gas is a distinct point in their favour. For arthritic cases this spa is admirably suited, owing to the exceptional facilities for orthopædic treatment which, thanks to the energy of the administration, have been abundantly provided. The bathing establishment is fitted up with every modern convenience; no one going to this spa need imagine for a moment that he is going to an antiquated or primitive establishment. He will find that everything is up to date so far as the arrangements for administering various treatments are concerned. There is a fine inhalatorium, a magnificently fitted orthopædic institute, a milk-cure establishment, and the hundred-and-one other appurtenances that belong to a properly managed and modern spa. The new Kurhaus is one of the best of its kind in Germany, while the bathing houses rival those of any other establishment with which we are acquainted.

The supervision of the place is in the hands of the Royal Board of Mines at Dortmund, which is directly controlled by the Minister of Commerce and Industry. Locally all arrangements are made by the Bade Verwaltung, at the head of which is a president, who is the manager of the Neusaltzwerk Mine, and who is assisted by an influential

The Weir Restaurant.

committee to which the various medical men who practice at the spa are attached. The taxes are extremely reasonable. During the summer months the Kur-tax is £1 for one person, with a charge of 5s. for each additional person in the family, and 2s. for each servant, medical men and their families being exempt from this, and also obtaining baths and treatment at reduced charges. The bathing charges vary from two to four marks (full price) and 3d. to 1s. (reduced rate). A

monthly ticket for the pump room costs 2s.; a single visit 1d. The charges made at the inhalatorium and for the taking of $x$-ray photographs are equally reasonable.

Accommodation.

Every care is taken, in the interests of the visitors, to control the public lodging-houses, and to supervise, generally, the arrangements for accommodation of patients. There are excellent, though by no means overawing hotels for those who wish to stay only for a couple of days; there are equally excellent pensions. Patients desiring to make a long stay are recommended to take private apartments, which can be readily obtained at a charge of from 7 to 75 marks per week, according to size and style, while catering can easily be arranged for at moderate charges. The restaurant in the Kurhaus is excellent, and the larger hotels have equally good restaurants attached. Visitors are recommended to familiarise themselves with

Bath-house I. (West Wing).

the official regulations with regard to the letting of apartments, and there has been established a special reference and inquiry department, where patients can obtain all information immediately on arrival. A special, and eminently satisfactory feature of the Spa is the "Johanniterasyl," which is a small hospital, pleasantly situated in a large park, where patients of small means can be re-

Kaiser Wilhelm Sprudel.

ceived and specially treated. Such patients must bring with them, when making application for admission to the committee (addressed Colonel Westwerdt, Oeynhausen), a certificate signed by a trustworthy authority testifying that they cannot afford private treatment, another certificate from their medical attendant prescribing a course of treatment at the spa, and a voucher guaranteeing the small charge made at the home. This charge is indeed merely nominal, being 1s. 2d. per day, for which the patient receives board and lodging, together with medical and bathing treatment. When the application has been granted, the applicant also receives a special return ticket to Oeynhausen at a much reduced fare.

Amusements.

The Bade Verwaltung has done everything possible to ensure that the visitor to the Spa shall not be troubled with ennui, but at the same time, with commendable foresight, it has desisted from embarking on the gigantic amusement schemes which are so regrettable a feature of some Continental establishments. The visitor to Oeynhausen may rest assured that he or she will not be inconvenienced through the relaxations provided for those who come to the little town as much for relaxation as in search of a cure. The main feature at Oeynhausen is that it is the nearest approach to home that a spa can offer; the whole place is, as it were, a gigantic "Liberty Hall." For those who delight in music and entertainment, there is always the Kurhaus and its excellent park, with fine promenades and an excellent band. Dances are given here and theatricals, with now and then special symphony concerts, which are much appreciated. In the Kur park there are several lawn-tennis grounds, and the children have been cared for in a special "Children's Playground." Good fishing is obtainable in the river, and rowing facilities are provided. In addition there is a fine reading-room where all the latest papers and literature are provided free of charge. The neighbourhood abounds in interesting walks, and those who wish to go further afield can find plenty of scope for planning excursions through the mountains, or in the woods. The charges for horse and carriage hire, regulated by the municipality, are very low, while the roads are excellent for walking or bicycling. Spots of interest in the neighbourhood can also be easily reached by train.

Doctors and Medical Attendants.

A word must be said in conclusion with regard to the medical attendance at the spa. There are many practitioners at Oeynhausen, and the visitor will be able to select his or her medical attendant according to choice. All the doctors are on the committee of management and are specialists in "Oeynhausen treatment." English and French are freely spoken, and the English visitor will find no difficulty whatever in making himself at home in what is, when everything is taken into consideration, one of the most ably managed and most attractive of German health-resorts. Those who desire further particulars about Oeynhausen Spa are recommended to write direct to the London agency, at 23 Old Jewry, E.C., where illustrated pamphlets in English, giving detailed information about treatment, accommodation, routes, the establishment itself, and the names of medical men in attendance at the baths, may be obtained.

## Figures and Tables

**Figure f1:**
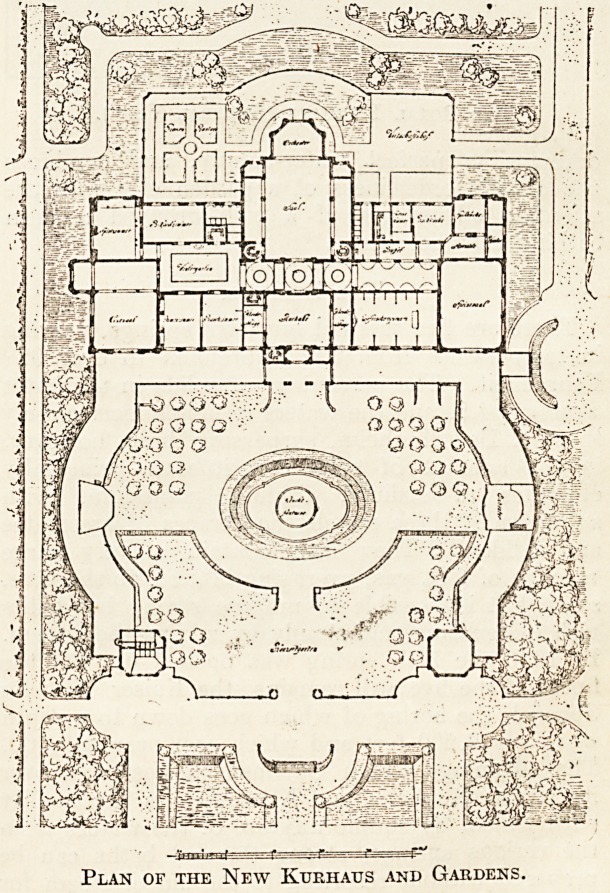


**Figure f2:**
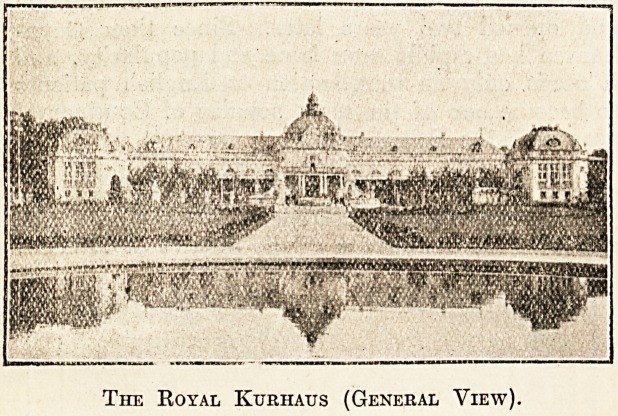


**Figure f3:**
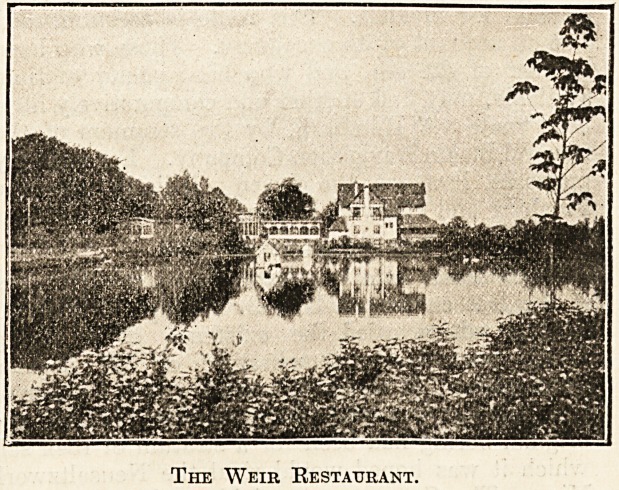


**Figure f4:**
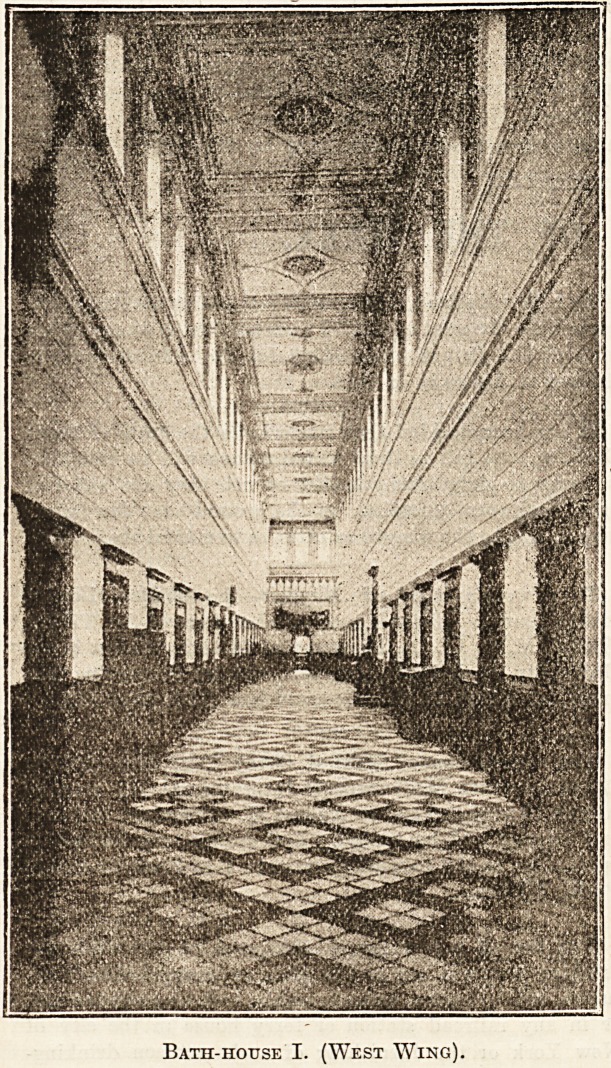


**Figure f5:**